# First principles exploration of structural, electronic, and optical properties of M_2_XT_2_ (M = Hf, Zr; X = C; T = O, F) MXenes for photovoltaic applications

**DOI:** 10.1039/d5na00673b

**Published:** 2025-11-19

**Authors:** Wassila Derafa, Iftikhar Ahmed, Asif Saeed, Nada Alhathlaul, Khawar Ismail, Hassan Ali, Calvyn T. Howells, Rasheed Ahmad Khera, Muhammad Faizan

**Affiliations:** a Department of Chemistry, College of Science, Jouf University Sakaka Aljouf 72341 Saudi Arabia; b Environmental and Public Health Department, College of Health Sciences, Abu Dhabi University P.O. Box 59911 Abu Dhabi United Arab Emirates; c Department of Physics, University of Narowal 51600 Pakistan; d Dipartimento di Matematica e Fisica “E. De Giorgi,” Università Del Salento Via Arnesano 73100 Lecce Italy; e Department of Chemistry, University of Agriculture Faisalabad 38040 Pakistan; f Department of Physics, University of Peshawar Peshawar 25120 Pakistan faizanstd@uop.edu.pk faizanjlu2022@jlu.edu.cn; g School of Materials Science and Engineering, Jilin University Changchun Jilin 130012 China

## Abstract

MXene compounds are promising candidates for energy-related applications because of their tunable electronic and surface properties. In this work, we explore structural, electronic, and optical features of M_2_XT_2_ (M = Hf, Zr; X = C; T = O, F) using density functional theory (DFT) within the WIEN2k computational framework. These compounds show non-magnetic behavior and exhibit a hexagonal geometry (space group# 194). Phonon dispersion analysis confirms that these compounds are dynamically stable. Hf_2_CO_2_ and Zr_2_CO_2_ exhibited indirect bandgaps of 1.1 and 1.12 eV, respectively, classifying them as solar absorber materials. Conversely, Hf_2_CF_2_ and Zr_2_CF_2_ are semimetals because of the small energy interband overlapping. Their optical properties were explained in terms of optical conductivity, loss parameter, extinction coefficient, refractive index, reflectivity, absorption coefficient, and dielectric function. Our findings indicate that M_2_XT_2_ materials are suitable for sustainable energy technologies, including photovoltaic cells and other optoelectronic applications.

## Abstract 2

MXene compounds are promising candidates for energy-related applications because of their tunable electronic and surface properties.

## Graphical abstract

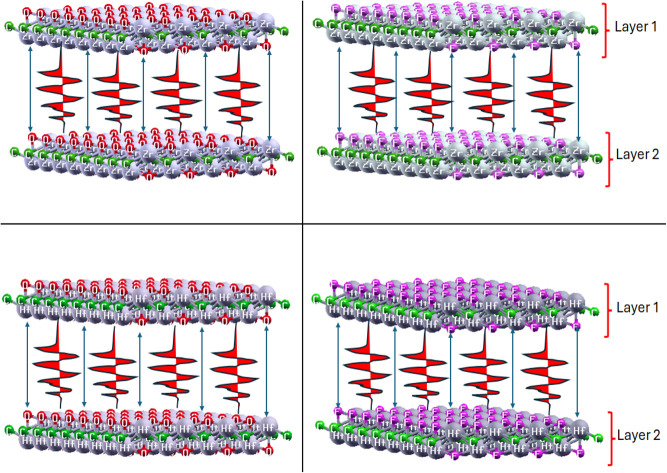

## 1. Introduction

The energy requirements increase day by day due to the large consumption driven by human activity.^1–4^ The world is grappling with a severe energy crisis because traditional fossil-fuel based resources are finite, contribute significantly to climate change and are often concentrated in politically unstable regions. This overreliance on fossil fuels has led to geopolitical tensions, environmental degradation, and economic vulnerabilities. Thus, researchers are trying to find new sustainable and eco-friendly energy sources that do not harm the environment.^5^ Solar energy stands out as one of the most promising solutions to today's energy and environmental challenges. It is abundant, renewable, cost-effective, and environmentally friendly. Through photovoltaic cells, solar panels convert sunlight directly into electricity, offering a clean and sustainable alternative to fossil fuels. Unlike conventional energy sources, solar power generates electricity without releasing harmful gases, helping to mitigate the effects of climate change. To keep up with the growing global demand for green energy, it has become essential to explore and develop novel materials that can enhance the performance of photovoltaic technologies and optoelectronic devices.

Over the last decade, two-dimensional (2D) materials have gained significant attention as fundamental building blocks for a wide range of applications, from electronic devices to energy storage and catalysis. The distinctive planar geometry of 2D materials allows for their integration into numerous technological devices.^6^ The optical properties of 2D materials indicate that they are suitable for solar energy absorption and transparent heat mirrors. The dimensions of nanocrystals and thin films significantly influence their electronic and optical behavior, leading to the development of innovative devices such as photodiodes, photovoltaic cells, and light-emitting diodes.^7^ The 2D graphene material discovered in 2004 ignited significant concern in two-dimensional (2D) materials.^8,9^ Its exceptional electronic, optical, and mechanical properties inspired researchers to explore further 2D materials,^10,11^ namely TMDCs^12^ and hexagonal BN.^13^

The novel family of 2D MXene nanomaterials discovered by the Gogotsi group in 2011 ^14,15^ with the potential to revolutionize various fields: water purification,^16–18^ biomedical fields,^19–21^ light-emitting diodes,^22^ catalysis,^23,24^ energy storage^25–27^ and electromagnetic applications.^21,28^ Moreover, they have demonstrated excellent results in the field of solar cell technology. The first stated MXene, titanium carbide (Ti_3_C_2_), emanates from Drexel University research.^15^ Their journey begins with MAX phases, three-dimensional layered materials with a specific formula M_*n*+1_AX_*n*_ (where M = Hf, Zr, Ti, X = N or C and A = Al, Si, S).^29^ A strong covalent bond is present between M and X, whereas a relatively weak bond exists between M and A, so the A layer can be removed by the etching method to form M_*n*+1_X_*n*_ as a 2D nanomaterial.^14^ MXenes are a class of exciting 2D materials represented by the general formula M_*n*+1_X_*n*_T_*x*_. M denotes an early transition metal, M = Hf, Zr, Ti, and X = N or C. n denotes an integer, typically extent from 1 to 4, that defines the number of M atoms presents in a single repeating unit within the MXene structure and T_*x*_ represents the surface terminations, which are essentially functionalized groups such as O, Br, I, and F attached to the surfaces of the atomic layers of MXenes.^30,31^ These terminations can influence the properties of the material.

Ti_3_C_2_T_*x*_ MXenes offer excellent features such as maximum carrier mobility, electrical conductivity, tunable work function, and transparency, which make them promising materials for photovoltaic cells.^32–34^ Starting in 2018, Ti_3_C_2_T_*x*_ was first incorporated into perovskite solar cells based on the photoactive layer of MAPbI_3_.^35^ Ti_3_C_2_T_*x*_ has been successfully integrated into different parts of solar cells, such as electrodes, hole and electron transport layers, as well as additives within these layers. Building on early MXene research, Naguib *et al.*^14,30,31^ expanded the scope to include other transition-metal carbides, particularly hafnium carbide Hf_2_C and zirconium carbide Zr_2_C. Their findings highlighted the strong potential of these materials for thermoelectric and optoelectronic applications. Upon oxidation, Hf_2_C and Zr_2_C transform into Hf_2_CO_2_ and Zr_2_CO_2_, which exhibit semiconducting behavior with tunable band gaps, making them promising candidates for photodetectors and related electronic devices.^36^ When functionalized with fluorine, these carbides form Zr_2_CF_2_ and Hf_2_CF_2_, which display semi-metallic properties with excellent electrical conductivity, positioning them as efficient charge transport layers or electrodes in next-generation devices.^37^

Most current studies depend on theoretical predictions of MXenes with mixed terminations (–O, –F, and –OH), which makes it challenging to determine the role of a single termination. One of the main gaps in this area is the absence of reliable synthesis routes for obtaining fully O-terminated (Hf_2_CO_2_ and Zr_2_CO_2_) or fully F-terminated (Hf_2_CF_2_ and Zr_2_CF_2_) structures. The novelty of the present work lies in addressing this limitation by focusing on such pure terminations and performing a direct comparative analysis. The class of M_2_XT_2_ MXenes, such as, Hf_2_CO_2_, Zr_2_CO_2_, Hf_2_CF_2_, and Zr_2_CF_2_ materials, has emerged as promising candidates with adjustable surface terminations, chemical stability, and diverse electronic characteristics. Density Functional Theory (DFT) serves as an effective tool to probe their electronic structure, optical behavior, and charge transport, providing valuable predictions where experiments face limitations. A systematic DFT investigation of these understudied materials clarifies how the surface termination groups oxygen and fluorine (O and F) and transition metal species hafnium and zirconium (Hf and Zr) influence their functional properties. The incorporation of relatively under-investigated MXene compounds Hf_2_CO_2_, Hf_2_CF_2_, Zr_2_CF_2_, and Zr_2_CO_2_ into solar cell designs shows strong promise for improving both device efficiency and stability. These materials stand out owing to their excellent electronic conduction, modulation in band gaps, and strong light-absorbing capabilities, all of which can enhance charge carrier transport and reduce recombination losses, thereby boosting photovoltaic performance. In this research work, DFT calculations within the WIEN2K framework are employed to investigate the structural, electronic, and optical properties of M_2_XT_2_ systems (M = Hf, Zr; X = C; T = O, F). The results reveal that both Hf_2_CO_2_ and Zr_2_CO_2_ possess an indirect bandgap of ∼1.15, making them highly suitable contenders for solar absorbers and photo-detection applications. In contrast, the fluorinated variants, Hf_2_CF_2_ and Zr_2_CF_2_, exhibit semimetallic behavior. Total and partial density of states (TDOS and PDOS) analyses confirm their favorable charge transport characteristics. Moreover, optical studies demonstrate strong absorption in the visible range, high reflectivity, and low energy loss. Collectively, these features highlight the considerable potential of these understudied MXenes for advancing next-generation solar cell technologies.

## 2. Computational method

The structural, electronic, optical, and thermoelectric features were calculated with the help of the computational tool, which is density functional theory (DFT).^38^ In the present work, we employed DFT based on the full potential linear augmented plane wave (FP-LAPW) approach that was implemented within the Wien2k computational code.^39^ In order to minimize interatomic forces and stabilize the structures, the crystal structures were optimized using the TB-mBJ approximation.^40,41^ The Trans and Blaha modified Becke–Johnson potential (TB-mBJ)^40^ was employed to determine the electronic and optical characteristics because the Perdew–Burke–Ernzerhof for solids (PBEsol) exchange–correlation functional analyses the ground state properties more precisely but underestimates the electronic band gap. In order to increase the band gap accuracy, the TB-mBJ potential has been used instead of the PBEsol approximation.^42^

The phonon dispersion curves were obtained using the Phonopy package, a reliable tool commonly employed for analyzing vibrational properties and assessing dynamical stability. Moreover, the electronic system's solution is assumed as circularly harmonic in the muffin-tin area and plane wave-like in the interstitial zone. The initial parameters in the reciprocal lattice were set as follows: the radial wave function is expanded to *l*_max_ = 10, the Gaussian parameter *G*_max_ = 12, and the product of the wave vector and muffin radius, *K*_MAX_*R*_MT_ = 10. A k-mesh of size 12 × 12 × 12, corresponding to 1000 k-points in the irreducible Brillouin zone, was selected as it ensures the convergence of the system's total energy. This is the upper threshold of the convergence criteria for achieving high precision results. The charge convergence criterion is set to 0.00001 Ry. In order to measure the frequency-dependent parameters, the Kramers–Kronig dispersion relation is used. Furthermore, the BoltzTraP code,^43^ based on the classical Boltzmann transport theory, has been utilized to calculate the transport properties using the converged total energy and the optimized electronic structures obtained with the TB-mBJ potential.^44^

## 3. Results and discussion

### 3.1 Structural properties

**Fig. 1:**
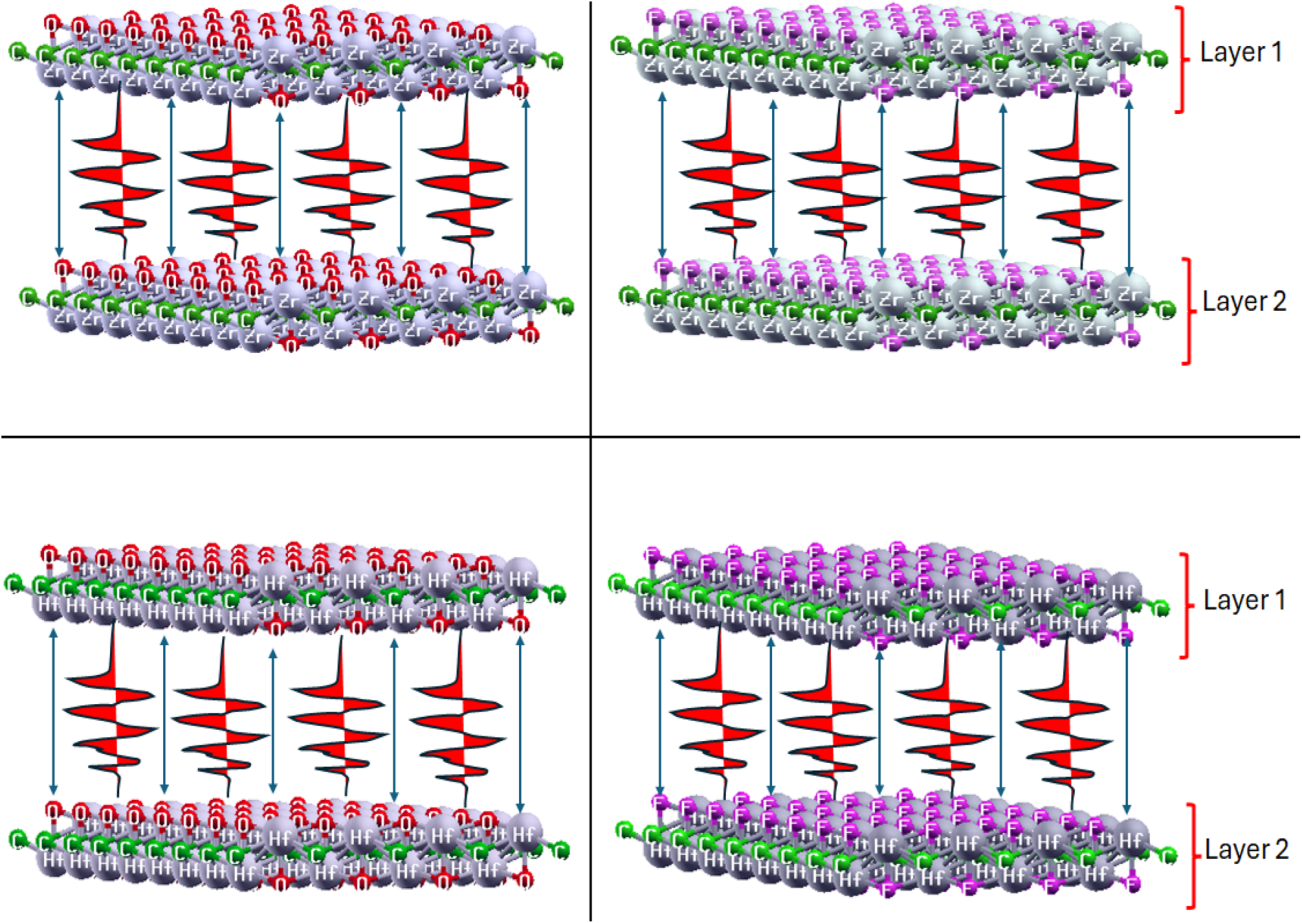
Layered structures of Zr_2_CO_2_, Hf_2_CO_2_, Zr_2_CF_2_ and Hf_2_CF_2_.

**Fig. 2:**
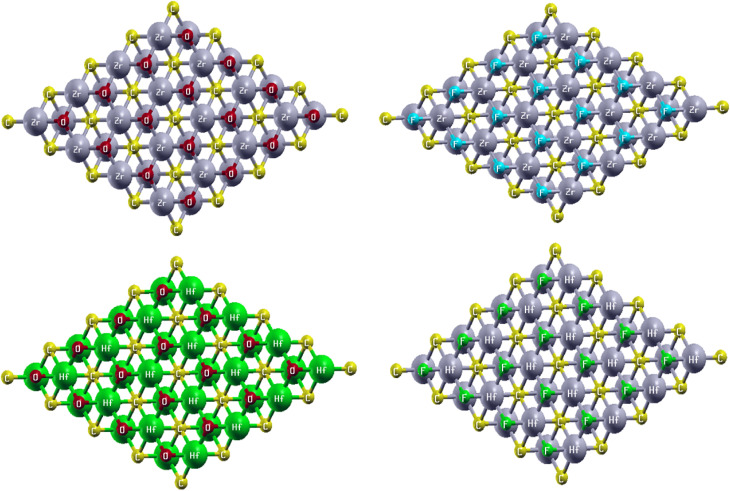
Top view of Zr_2_CO_2_, Hf_2_CO_2_, Zr_2_CF_2_ and Hf_2_CF_2_.

The basic information of a compound can be described from its crystal structure. The MXene under study, with the formula M_2_XT_2_ (where M = Hf and Zr, X = C, and T = O and F), reveals a hexagonal close-packed (hcp) layered crystal lattice, with carbon (C) atoms positioned at the octahedral sites between the layers. The conduction gap from layer 1 and layer 2 is the van der Waals gap.^43^ The layers are arranged along the c-axis with van der Waals gaps between them, which makes exfoliation into single- and few-layer nanosheets relatively easy, as illustrated in Fig. 1. The layer of carbon atoms (C) is sandwiched inside the Zr/Hf layers and surface termination T is attached at the upper and lower surfaces of the Zr/Hf layers represented as a single layered structure view, as shown in Fig. 1, and the top view of the hcp crystal structure is given in Fig. 2.

In the top views of the understudied MXenes, the red balls indicate oxygen atoms and light blue and light green balls represent fluorine atoms, whereas the yellow balls indicate carbon atoms. The green and grey balls indicate hafnium atoms, and the grey balls indicate zirconium atoms in different structures. The metal atoms Zr/Hf form a closely packed hexagonal lattice, resembling a honeycomb pattern similar to graphene, and serve as the backbone of the MXene layers. The carbon atoms occupy the centers of these hexagons, bonded to six surrounding metal atoms. This arrangement involves a combination of metallic and covalent bonds. The surface termination atoms (T) are attached to the MXene layers, influencing conductivity, hydrophilicity, reactivity, and overall chemical behavior. The hexagonal lattice is interpreted by parameters *a*, *b*, and *c*, with *a* and *b* being equal (*a* = *b* = 3.3 Å) and *c* varying depending on the specific MXene composition (*e.g.*, 18.52 Å for Hf_2_CO_2_, 19.58 Å for Hf_2_CF_2_, 17.99 Å for Zr_2_CO_2_, and 19.05 Å for Zr_2_CF_2_), as shown in Table 1. The lattice angles *α* and *β* are 90° and *γ* is 120°, as determined by X-ray diffraction techniques. Their lattice parameters were calculated using TB-mBJ potential exchange–correlation functionals.^45^Eqn (1) gives the formation energy Δ*E*_f_ of terminated MXenes (M_2_CT_2_), determined from the reference energies of their constituent elements (M = Hf/Zr, C, T = O/F). Table 1 indicates that the understudied MXenes possess negative formation energy, confirming their thermodynamic stability and suggesting their potential for experimental synthesis. The bulk modulus and its derivative are obtained by fitting the Murnaghan equation of state (EOS) (2).^36^1
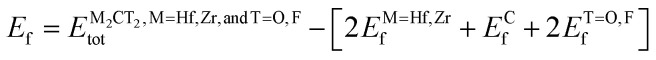
2
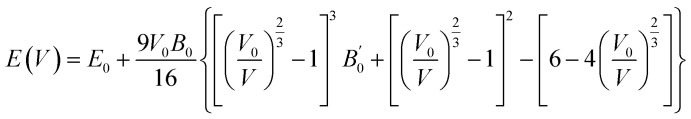


**Table 1:** Lattice parameters *a*, *b*, *c* (Å), band gap *E*_g_ (eV), and formation energy Δ*E*_f_ of Zr_2_CO_2_, Hf_2_CO_2_, Zr_2_CF_2_ and Hf_2_CF_2_

Compound	*a* = *b* (Å)	*c* (Å)	*E*_g_ (eV)	Δ*E*_f_
Zr_2_CO_2_	3.3	17.99	1.15	−9775.6944
Hf_2_CO_2_	3.3	18.52	1.16	−20 362.7283
Zr_2_CF_2_	3.3	19.05	0	−10 252.8254
Hf_2_CF_2_	3.3	19.58	0	−20 838.1861

### 3.2 Electronic properties

The electronic characteristics of an understudied material are defined by the band gap structure and the density of states.^46^

#### 3.2.1 Band structure

**Fig. 3:**
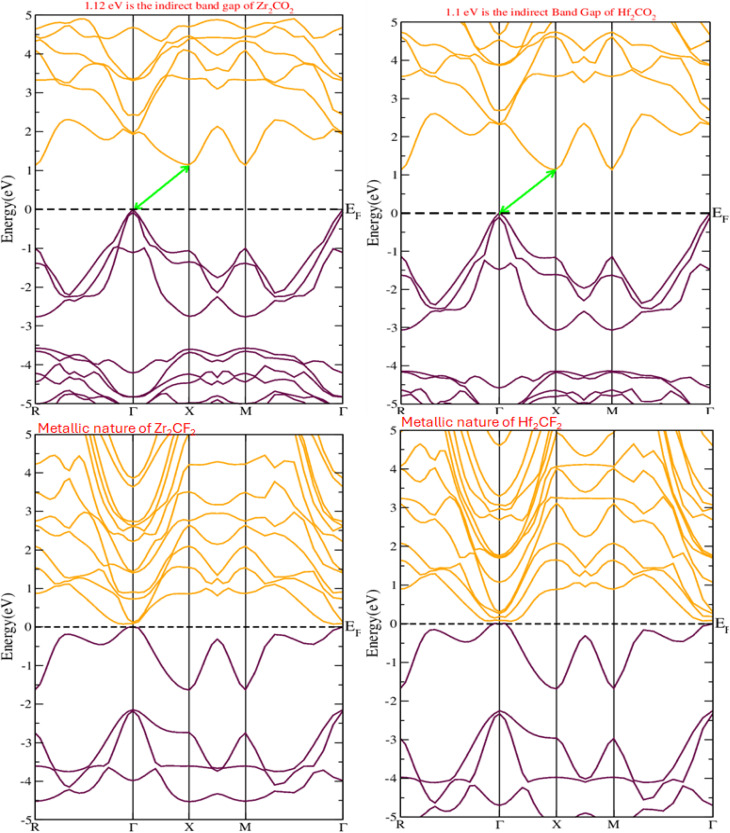
Band structures of Zr_2_CO_2,_ Hf_2_CO_2_, Zr_2_CF_2_, and Hf_2_CF_2_.

The electronic properties of the under-investigated material were examined using the band structure, which provides information about its bandgap type and classification as a semiconductor, insulator, or metal.^47^ This study employed the Perdew–Burke–Ernzerhof generalized gradient approximation (PBEsol-GGA) to investigate the electronic band structure (EBS) of hexagonal M_2_XT_2_ (M = Hf, Zr; X = C; T = O, F) MXenes as shown in Fig. 3. The EBS outlines energy regions where electrons can (energy bands) and cannot (bandgap) exist. The Fermi level, represented by dashed lines, separates the conduction and valence bands at zero energy. Energy levels above the Fermi level belong to the conduction band, while those below constitute the valence band. By analyzing the band structure within a −5 to 5 eV energy range, it was determined that Hf_2_CO_2_ and Zr_2_CO_2_ exhibit indirect bandgaps, characteristic of semiconductors. Conversely, Hf_2_CF_2_ and Zr_2_CF_2_ show a small energy overlap between the minimum and maximum of the conduction and valence bands, indicating semimetal behavior. Because of this small energy overlap (band gap) of Hf_2_CF_2_ and Zr_2_CF_2_ materials, the photo-generated electrons and holes will likely rejoin rapidly in the visible portion before they can participate in photocatalytic reactions. So, they may not be ideal for photocatalytic applications because the recombination rate of photo-induced charge carriers is higher. Therefore, for use in solar cells, the surface chemistry of M_2_XT_2_ MXenes plays a crucial role. Oxygen-terminated materials such as Hf_2_CO_2_ and Zr_2_CO_2_ have a semiconducting nature, which makes them effective at absorbing light and creating electron–hole pairs. Conversely, fluorine-terminated MXenes such as Hf_2_CF_2_ and Zr_2_CF_2_ are highly conductive, allowing them to efficiently move photo-generated charges to electrodes. The calculated band gaps for Hf_2_CO_2_ and Zr_2_CO_2_ are 1.1 eV and 1.12 eV, respectively, so both materials are suitable for visible-light-driven photocatalysis due to their potential to absorb visible light. Furthermore, Hf_2_CO_2_ and Zr_2_CO_2_ possess indirect band gaps situated at distinct k-points. This characteristic can help to reduce the rejoin of photo-generated hole–electron pairs. These results are approximately consistent with previously reported results.^48–52^

#### 3.2.2 Density of states

**Fig. 4:**
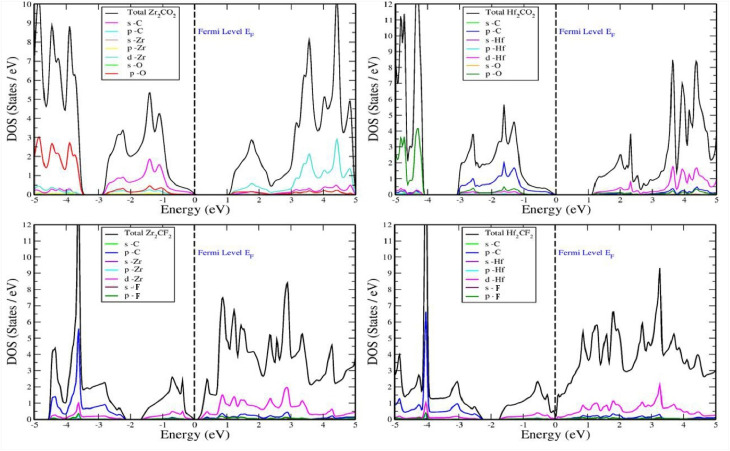
Total density of states and partial density of states of (top left panel) Zr_2_CO_2_, (top right panel) Hf_2_CO_2_, (bottom left panel) Zr_2_CF_2_ and (bottom right panel) Hf_2_CF_2_.

Density of states (DOS) analysis was performed to investigate individual electron contributions and the composition of hybrid states. This characteristic is important for determining whether a material is metallic or semiconducting and provides detailed insights into its band structure.^53^ To complement the electronic band structure calculations, the total density of states (TDOS) and partial density of states (PDOS) for Zr_2_CO_2_, Hf_2_CO_2_, Zr_2_CF_2_, and Hf_2_CF_2_ were computed and are presented in Fig. 4. The energy range for both TDOS and PDOS was set from −5 to 5 eV, and zero energy illustrated by a vertical dashed line indicates the Fermi level. Maximum occupied TDOS states were found to be 13.5, 18, and 15 states per eV for Hf_2_CO_2_, Hf_2_CF_2_, and Zr_2_CF_2_, respectively, in the valence band, while Zr_2_CO_2_ exhibited a maximum occupied state of 10.7 states per eV in the conduction band. The electronic band structure results are aligned with these results.

To elucidate orbital contributions, PDOS analysis was conducted for each atom. Additionally, PDOS calculations were performed for all understudied materials to correlate band characteristics with surface group types and geometries, as depicted in Fig. 4. In Zr_2_CO_2_, oxygen and zirconium atoms significantly impact the TDOS, particularly within the valence band. The contributions of oxygen and zirconium are highlighted to represent the Fermi level's proximity to the valence band. A more detailed analysis of the PDOS reveals that the valence band is composed of carbon s-states, oxygen p-states, and zirconium p- and d-states. This combination of states contributes to the Fermi level's position near the valence band, as depicted in Fig. 4(a) and (b), which illustrates the electronic structure of Hf_2_CO_2_. The Fermi level is positioned near the valence band, primarily because of the significant contributions from the p-states of carbon and oxygen and the d-states of hafnium. Conversely, the contributions from other atomic states are less. While oxygen p-states display a notable participation in the valence band, oxygen's overall contribution to the TDOS is significant, as depicted in Fig. 4(b). For oxygen-terminated MXenes Hf_2_CO_2_ and Zr_2_CO_2_, the density of states (DOS) displays a bandgap of 1.1 eV and 1.12 eV, confirming their semiconducting character. The PDOS shows that this gap originates from the interaction between metal Hf and Zr d-orbitals and oxygen p-orbitals, giving rise to electronic structures favorable for light absorption. In Zr_2_CF_2_, zirconium, carbon, and fluorine atoms significantly impact the electronic band structure of both the valence and conduction bands near the Fermi level, as observed in the TDOS. A closer examination of the PDOS shows that zirconium d-states primarily contribute to the DOS in the conduction band and carbon p-states contribute to the DOS within the valence band. At very high magnification, the p-states of zirconium, carbon, and fluorine are also seen to contribute to the conduction band suggesting that these elements may also have conductive properties as shown in Fig. 4(c) In Hf_2_CF_2_, hafnium, carbon, and fluorine atoms significantly influence the electronic structure of both the valence and conduction bands around the Fermi level, as observed in the TDOS. A more detailed examination of the PDOS reveals that hafnium d-states dominate the DOS in both the valence and conduction bands around the Fermi level and carbon p-states contribute to the DOS within the valence band, as shown in Fig. 4(d).^54^ Conversely, fluorine-terminated MXenes Hf_2_CF_2_ and Zr_2_CF_2_ exhibit semi-metallic behavior, as evidenced by a DOS at the Fermi level. So, the PDOS highlights significant contributions from carbon p-orbitals and metal Hf and Zr d-orbitals around the Fermi energy, resulting in high electrical conductivity. An important distinction also arises between Hf- and Zr-based MXenes. Due to lanthanide contraction, Hf atoms are slightly smaller than Zr, leading to stronger orbital overlap and minor shifts in the electronic structure.^55^ As a result, Hf-containing MXenes often present a marginally larger bandgap or a shifted Fermi level compared to Zr. These variations, tied to the role of metal Hf and Zr d-orbitals in the DOS, influence optical transition energies and consequently affect the materials’ potential efficiency in photovoltaic applications.

### 3.3 Optical properties

MXene materials exhibit unique optical properties that are necessary for the advancement of photovoltaic cells and optoelectronic devices. The optical behavior of M_2_XT_2_ MXenes (where M = Hf, Zr, X = C and T = O, F) is influenced by the light frequency.^52^ The dielectric function *ε*(*ω*) = *ε*_1_(*ω*) + *ε*_2_(*ω*) is an optical parameter that characterizes a material's response to an electric field. The real part *ε*_1_(*ω*) provides information about material polarization and the imaginary part *ε*_2_(*ω*) relates to light absorption, determined using the Kramers–Kronig relation.^56^3
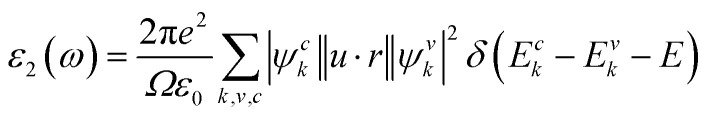
In the above expression, *u* denotes the polarization vector, *Ω* represents the unit cell volume, and *e* represents the elementary charge. The parameter *ω* corresponds to the frequency of the incoming photon. The wave functions *ψ*^*c*^_*k*_ and *ψ*^*v*^_*k*_ describe the electronic states in the conduction and valence bands with their associated energies given by *E*^*c*^_*k*_ and *E*^*v*^_*k*_. The Dirac delta function *δ*(*E*^*c*^_*k*_ − *E*^*v*^_*k*_ − *E*) enforces the principle of energy conservation during optical transitions. Finally, the term *u*·*r* corresponds to the dipole (or momentum) matrix element.4
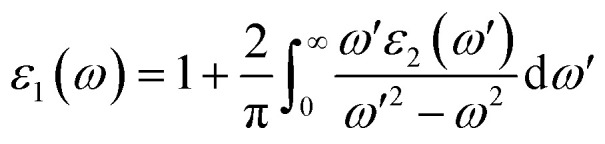


The refractive index, extinction coefficient, optical conductivity, absorption spectrum, reflectivity, and loss parameter can be measured from a given set of mathematical equations.^44^5
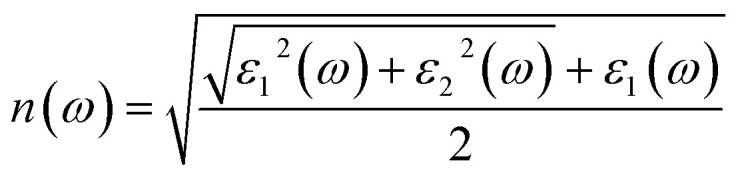
6
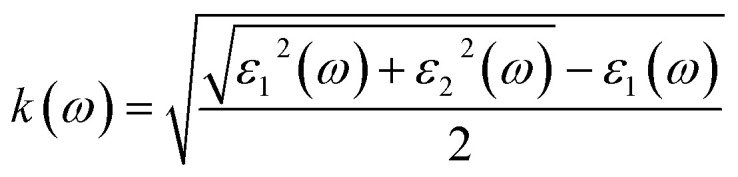
7
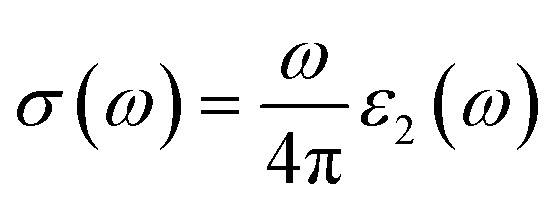
8
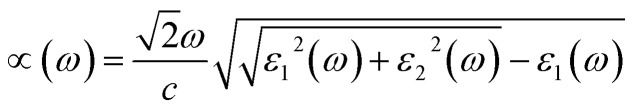
9
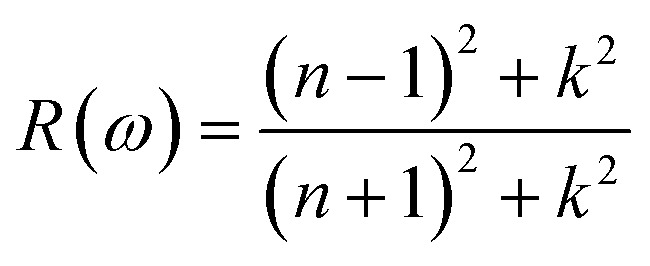
10
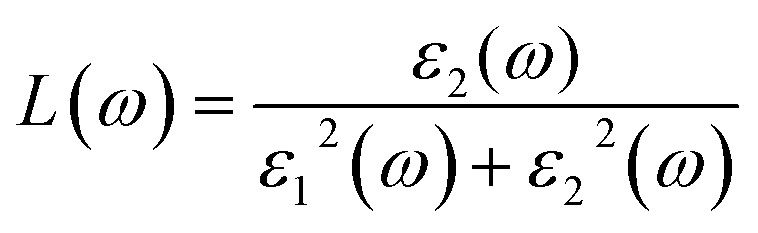


**Fig. 5:**
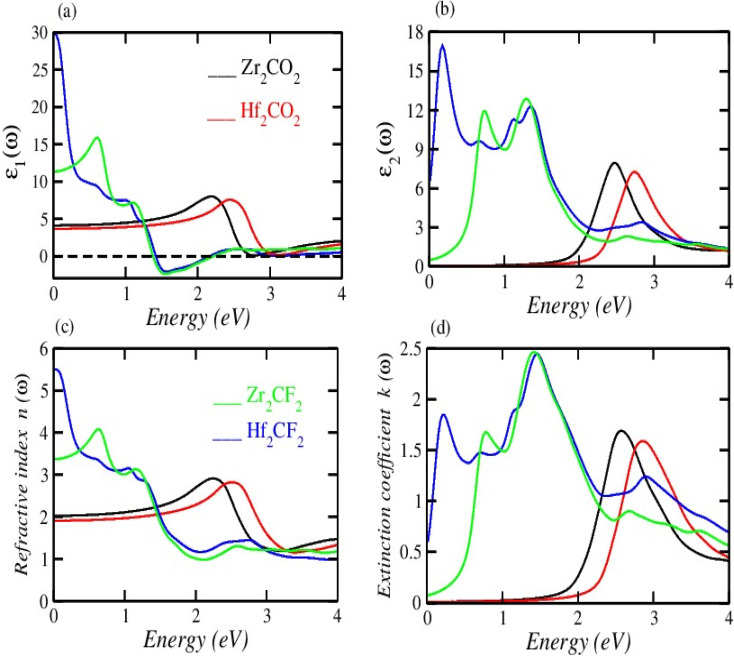
(a) Real part *ε*_1_(*ω*) and (b) imaginary part *ε*_2_(*ω*) of the dielectric function, (c) refractive index *n*(*ω*) and (d) extinction coefficient *k*(*ω*).

**Fig. 6:**
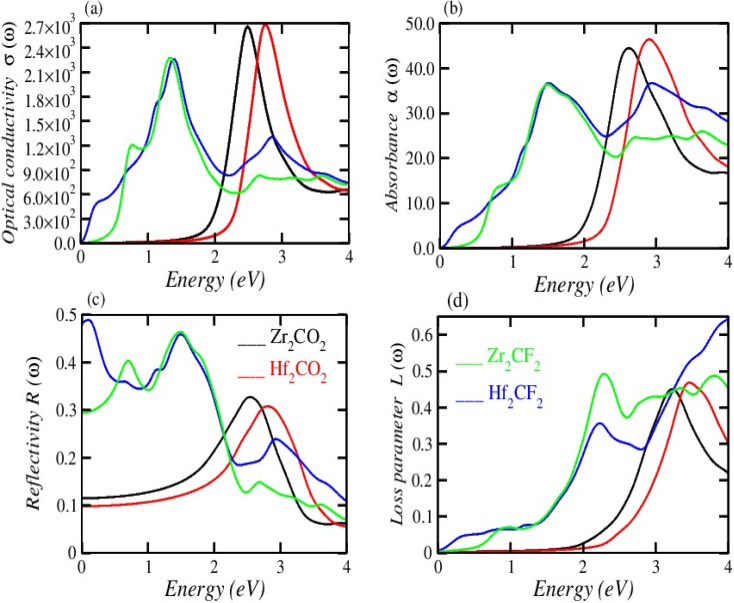
(a) Optical conductivity *σ*(*ω*), (b) absorption coefficient *α*(*ω*), (c) reflectivity *R*(*ω*) and (d) loss parameter *L*(*ω*).

Fig. 5(a) illustrates the measured real part of the studied material with a photon energy range of 0 to 4 eV. The static real part *ε*_1_(*ω*) can be determined for the M_2_XT_2_ compounds (where M = Hf or Zr, X = C, and T = O or F). Specifically, Hf_2_CO_2_, Zr_2_CO_2_, Hf_2_CF_2_, and Zr_2_CF_2_ exhibit static *ε*_1_(*ω*) values of 3.5, 4, 30, and 12, respectively. Notably, Hf_2_CF_2_ exhibits the highest static *ε*_1_(*ω*), suggesting superior dielectric properties compared to the other materials. Higher *ε*_1_(*ω*) values are generally associated with reduced charge carrier recombination rates. The *ε*_1_(*ω*) values of Hf_2_CO_2_, Zr_2_CO_2_, Hf_2_CF_2_ and Zr_2_CF_2_ decrease with increasing photon energy. Specifically, *ε*_1_(*ω*) reaches zero at 2.8, 3, 1.51, and 1.5 eV for Hf_2_CO_2_, Zr_2_CO_2_, Hf_2_CF_2_ and Zr_2_CF_2_, respectively. While *ε*_1_ is initially constant and then decreases for Zr_2_CO_2_ and Hf_2_CO_2_, *ε*_1_(*ω*) initially increases and then decreases for Zr_2_CF_2_, whereas Hf_2_CF_2_ shows a rapid decline. These findings suggest that the investigated materials Hf_2_CO_2_ and Zr_2_CO_2_ have larger *ε*_1_(*ω*) in the visible region, which makes them excellent candidates for photovoltaic cells to store maximum energy with reduced charge carrier recombination rates as compared to Hf_2_CF_2_ and Zr_2_CF_2_. The imaginary part *ε*_2_(*ω*) is associated with electronic transitions between energy bands. Fig. 5(b) illustrates the imaginary part *ε*_2_(*ω*) revealing absorption characteristics for each material. Zr_2_CO_2_ and Hf_2_CO_2_ exhibit zero absorption in the energy range of 0 to 1.3 eV, indicating transparency in this portion. Conversely, non-zero *ε*_2_(*ω*) values signify absorption within specific energy ranges. Peak absorption for Hf_2_CO_2_, Zr_2_CO_2_, Hf_2_CF_2_, and Zr_2_CF_2_ occurs at 2.9 eV, 2.5 eV, 0.2 eV, and 1.35 eV, respectively. All of these understudied materials show excellent absorption in the visible portion, which helps them to use these materials in photovoltaic cells. Additional optical features, such as the reflectivity parameter, optical conductivity, reflectivity, *etc.*, can be derived from *ε*_2_(*ω*). The refractive index is a fundamental optical property that quantifies the fraction of the speed of light in a vacuum to the speed of light within a material. This parameter influences light refraction and transmission properties. For the studied material, the values of static refractive index are 1.9 for Hf_2_CO_2_, 2.0 for Zr_2_CO_2_, 5.5 for Hf_2_CF_2_, and 3.4 for Zr_2_CF_2_, as shown in Fig. 5(c). Generally, the refractive index initially increases and then followed by a decrease with increasing energy for Zr_2_CO_2_, Zr_2_CF_2_, and Hf_2_CO_2_. In contrast, Hf_2_CF_2_ shows a more abrupt decline. Across all compounds, the refractive index is higher at lower energies and decreases progressively at higher energies. The extinction coefficient *k*(*ω*) is closely comparable to the imaginary part and provides information about light absorption within a material. The mathematical relationship between these parameters is expressed as *ε*_2_(*ω*) = 2*nk*(*ω*).^57,58^Fig. 5(d) illustrates the *k*(*ω*) as a function of energy. Similar to *ε*_2_(*ω*), *k*(*ω*) is zero for Zr_2_CO_2_ and Hf_2_CO_2_ in the energy range of 0 to 1.3 eV, indicating transparency in this region. Non-zero *k* values correspond to light absorption. The static extinction coefficients for Hf_2_CO_2_ and Zr_2_CO_2_ are zero, while those for Hf_2_CF_2_ and Zr_2_CF_2_ are 0.7 and 0.1, respectively. The extinction coefficient increases with the increase of energy to reach peak values of 2.4 for Zr_2_CF_2_, 2.46 for Hf_2_CF_2_, 1.7 for Zr_2_CO_2_, and 1.63 for Hf_2_CO_2_. The *k*(*ω*) and *ε*_2_(*ω*) of the understudied material show approximately similar results in the visible region. Optical conductivity, *σ*(*ω*), describes how well a material can carry an electric current when it interacts with light. It quantifies the connection between incident light and the induced electrical current within the material.^59,60^ Optical conductivity is zero for all the understudied compounds under static conditions, as shown in Fig. 6(a). In the visible spectrum, both Zr_2_CO_2_ and Hf_2_CO_2_ exhibit optical conductivities of 2.7 × 10^3^ Ω^−1^ m^−1^ at 2.5 eV and 2.7 eV, respectively, while Zr_2_CF_2_ and Hf_2_CF_2_ show conductivities of 2.25 × 10^3^ Ω^−1^ m^−1^ at 1.4 eV and 1.41 eV, respectively. Zr_2_CO_2_ and Hf_2_CO_2_ are suitable for solar cells in the visible region because they show excellent optical conductivity as compared to Zr_2_CF_2_ and Hf_2_CF_2_. The conductivity plots reveal peaks and valleys. Although Zr_2_CO_2_ and Hf_2_CO_2_ compound groups exhibit a comparable peak within the studied energy range, overall, photon absorption enhances the electrical conductivity of these materials. Fig. 6(b) presents the investigated compounds' absorption coefficient spectra as a photon energy function. These data provide insights into the materials' light absorption capacity, including their potential for solar energy conversion. The absorption coefficient *α*(*ω*) measures how strongly a material absorbs light. It exhibits similar behavior to the *ε*_2_(*ω*) and is mathematically related to *k*(*ω*) through the equation *α*(*ω*) = 4π*k*/*λ*.^61,62^ The absorption coefficient initially has a zero value and progressively increases with rising energy values. In the visible spectrum, Hf_2_CO_2_, Zr_2_CO_2_, Hf_2_CF_2_, and Zr_2_CF_2_ display absorption coefficients of 46.7 m^−1^, 45.0 m^−1^, 36.2 m^−1^, and 36.2 m^−1^, respectively, at energies of 3.0 eV, 2.5 eV, 1.5 eV, and 1.5 eV. Consequently, Zr_2_CO_2_ and Hf_2_CO_2_ exhibit superior light-absorbed capabilities in the visible portion compared with Hf_2_CF_2_ and Zr_2_CF_2_. The amount of light that bounces off from a material's surface is measured by the reflectance parameter *R*(*ω*). Fig. 6(c) illustrates the studied compounds' reflectance coefficient *R*(*ω*). The static reflectance values *R*(0) for Hf_2_CO_2_, Zr_2_CO_2_, Hf_2_CF_2_, and Zr_2_CF_2_ are 0.1, 0.12, 0.49, and 0.29, respectively. Reflectance generally decreases with increasing energy.^63,64^ In the visible region at 3.3 eV, the minimum reflectance coefficients for Hf_2_CO_2_, Zr_2_CO_2_, Hf_2_CF_2_, and Zr_2_CF_2_ are 0.22, 0.11, 0.16, and 0.12, respectively. These results indicate that Zr_2_CO_2_ and Zr_2_CF_2_ reflect the minimum light in the visible portion at 3.3 eV, compared to Hf_2_CO_2_ and Hf_2_CF_2_. The loss parameter, *L*(*ω*), quantifies the energy dissipated when light interacts with a material. This energy loss can occur through various mechanisms, including dispersion, heating, or scattering. Energy loss is zero under the static conditions *L*(0). The loss parameter is zero in the visible region in the energy range of 1.3 to 1.7 eV for Hf_2_CO_2_ and Zr_2_CO_2_ as compared to Hf_2_CF_2_ and Zr_2_CF_2_. So, Hf_2_CO_2_ and Zr_2_CO_2_ can be used in photovoltaic cells in the energy range of 1.3 to 1.7 eV. However, the loss parameter increases with higher energy levels, as illustrated in Fig. 6(d).^54^Table 2 represents the calculated values of optical parameters under static conditions.

**Table 2:** Calculated optical properties of M_2_XT_2_ MXenes (M = Hf, Zr, X = C and T = O, F) obtained using the PBEsol approximation. All values are calculated at zero frequency

Compound	*ε*_1_(0)	*ε*_2_(0)	*n*(0)	*k*(0)	*σ*(0), Ω^−1^ m^−1^	*α*(0), m^−1^	*R*(0)	*L*(0)
Zr_2_CO_2_	4	0	2.0	0	0	0	0.12	0
Hf_2_CO_2_	3.5	0	1.9	0	0	0	0.1	0
Zr_2_CF_2_	12	0.5	3.4	0.1	0	0	0.29	0
Hf_2_CF_2_	30	6.6	5.5	0.7	0	0	0.49	0

### 3.4 Phonon dispersion

**Fig. 7:**
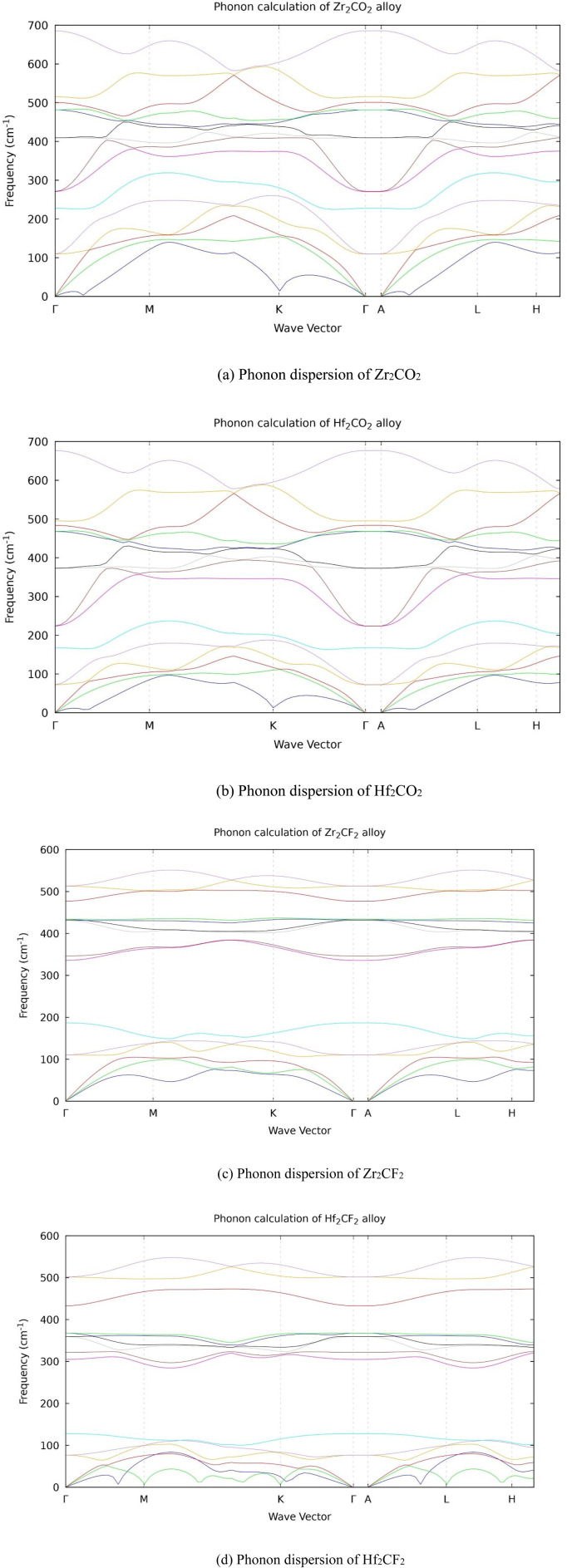
(a) Phonon dispersion of Zr_2_CO_2_. (b) Phonon dispersion of Hf_2_CO_2_. (c) Phonon dispersion of Zr_2_CF_2_. (d) Phonon dispersion of Hf_2_CF_2_.

The phonon spectra of Zr_2_CO_2_, Hf_2_CO_2_, Zr_2_CF_2_, and Hf_2_CF_2_ show no imaginary frequencies (negative frequency), confirming their dynamical stability. This absence of unstable vibrational modes ensures the structural robustness of the crystals. Phonon dispersion relations were determined along the high-symmetry paths of the Brillouin zone, with k-points serving as reference vectors. These dispersion curves, which display vibrational frequencies against the wave vector, provide essential information about phonon transport and their coupling with electrons and photons. The maximum phonon frequency range is ∼500–525 cm^−1^ for fluorinated and ∼670–680 cm^−1^ for oxygen-terminated MXenes, as depicted in Fig. 7, reflecting their structural stability and bonding strength.

## 4. Conclusions

This study investigated the physical properties of Hf_2_CO_2_, Zr_2_CO_2_, Hf_2_CF_2_, and Zr_2_CF_2_ for potential applications in photovoltaics. The data of formation energies and phonon calculation analyses demonstrate that these compounds are dynamically stable, suggesting they are suitable candidates for experimental synthesis. The results indicate that Hf_2_CO_2_ and Zr_2_CO_2_ possess indirect bandgaps of 1.1 eV and 1.12 eV, suggesting them as excellent semiconductor materials. In contrast, Hf_2_CF_2_ and Zr_2_CF_2_ exhibit small energy overlapping valence and conduction bands, characteristic of semimetals. However, oxygen-terminated compounds such as Hf_2_CO_2_ and Zr_2_CO_2_ have a semiconducting electronic structure that makes them excellent light absorbers, capturing photons and generating electron–hole pairs. In contrast, fluorine-terminated compounds, such as Hf_2_CF_2_ and Zr_2_CF_2_, are highly conductive, allowing them to serve as efficient charge transport layers or electrodes that swiftly move the photo-generated carriers to the external circuit. Moreover, the understudied materials demonstrate promising optical properties in the visible region, including high absorption coefficients of 46.7 m^−1^ and 45.0 m^−1^ for Hf_2_CO_2_ and Zr_2_CO_2_, reflectivity, and refractive index, coupled with low energy loss. These attributes show that understudied materials are excellent applicants for photovoltaic cells. Overall, this study provides a theoretical base for exploring the applicable implementation of these materials in advanced technological devices. However, future research on MXenes should move beyond the recent study of idealized MXene structures to examine the role of defects such as oxygen, bromine and fluorine, variation in surface terminations (–O, –Br, –I, and –F), and external influences such as mechanical strain, applied stress, and shifts in temperature. Integrating these factors with advanced computational methods such as hybrid functional (HSE) and DFT+U can improve the accuracy of electronic property predictions. Such efforts in defect engineering and external modulation will be vital for realizing the full potential of MXenes in next-generation sustainable technologies.

## Conflicts of interest

The authors disclose that they have no financial interests or personal connections that might have affected the research reported in this paper.

## Data Availability

Data will be made available upon reasonable request.
